# Alert for SARS-CoV-2 infection caused by fecal aerosols in rural areas in China

**DOI:** 10.1017/ice.2020.114

**Published:** 2020-04-07

**Authors:** Xiujuan Meng, Xun Huang, Pengcheng Zhou, Chunhui Li, Anhua Wu

**Affiliations:** Xiangya Hospital of Central South University, Changsha, Hunan Province, China

*To the Editor*—On March 11, 2020, The World Health Organization (WHO) director declared that >118,000 COVID-19 cases had been confirmed in 114 countries, that 4,291 people had lost their lives, and that COVID-19 could be characterized as a worldwide pandemic.^1^ The virus causing COVID-19, designated as severe acute respiratory syndrome coronavirus 2 (SARS-CoV-2), is closely related to SARS-CoV.^2^ In 2003, a SARS-CoV outbreak at Amoy Gardens in Hong Kong led to 329 confirmed cases of infection and 42 deaths.^3^ Subsequent studies suggested that the plumbing and ventilation systems at Amoy Gardens interacted to allow transmission of the SARS virus and that high concentrations of viral aerosols in the plumbing were the primary mode of transmission in this outbreak. Test results indicated that the hydraulic action caused by flushing toilets generated huge quantities of aerosols in vertical sewer pipes or sanitary risers.^4^

Recent studies found that SARS-CoV-2 can be detected in feces and urine of COVID-19 cases, especially the asymptomatic cases.^5^ SARS-CoV can persist in feces from infected people for as long as 4 days, and SARS-CoV-2 may persist in feces longer. Based on these characteristics, SARS-CoV-2 is prone to cause outbreaks in the community, particularly in rural areas. Excreta treatment in scattered rural areas is generally decentralized and self-processing. In concentrated areas, residents mainly use flush toilets, which can generate huge quantities of aerosols; the ventilation and plumbing systems in these places are not effective for maximal hygiene. The feces may form high concentrations of viral aerosols that travel through the air to cause infection.^6^

To prevent the spread of fecal aerosols, we recommend the following points. First, to avoid widespread viral aerosols in concentrated areas, state-of-the-art ventilation and plumbing systems should be constructed and maintained. Flouring half liter of water into each bathroom floor drain should be done weekly. In addition, the toilet lid should be covered when flushing the toilet to prevent aerosolization, and the toilet lid should be wiped with a disinfectant after flushing the toilet. Second, a safety program for environmental monitoring and feedback is an effective way to prevent the spread of SARS-CoV-2. Be alert to sewer gas, unusual noises, or bubbles in pipes and toilets, and respond immediately.^4^ Third, 3-segment septic-tank toilets and biogas tank toilets are the main sanitary toilets used in rural areas, and more effective raw sewage management should be explored in these areas. Another important aspect is natural ventilation, which can reduce viral density and is the most effective measure to reduce the risk of airborne contagion.^7^ By managing the feces of COVID-19 patients, we can effectively minimize the risks of viral spread in the community. Although the COVID-19 is described as a pandemic, we believe that our efforts can render this pandemic controllable.
